# Sexting in young university of the colombian caribbean, a comparative study between male and female

**DOI:** 10.1192/j.eurpsy.2021.1044

**Published:** 2021-08-13

**Authors:** E.P. Ruiz Gonzalez, M.N. Muñoz Argel, M.J. Arcos Guzman, A.M. Romero Otalvaro, D. Diaz Reyes

**Affiliations:** Psychology, Universidad Pontificia Bolivariana, Monteria, Colombia

**Keywords:** Sexting, Gender, Young

## Abstract

**Introduction:**

Sexting is sending / forwarding erotic-sexual content voluntarily through technological devices and / or the internet. (Fleschler-Peskin, 2013). Real Participation (RPS), Active Disposition (ADS) and Emotional Expression (EES) was studied.

**Objectives:**

Compare sexting in two groups of participants: female and male

**Methods:**

Comparison of data means measured by the Cronbach alpha sexting behavior scale α = 0.92, (Chacon-Lopez, et al, 2016). Sample N = 900 (447 female and 453 male)

**Results:**

The ADS and RPS decrease between 18 to 20 years old and increase between 20 to 22 years old. EES decreases when increasing age, except in 20 years old. Applying multiple regression analysis, control variable sex and reference group age 18 old, presents statistically significant difference, excepting 19 years old in EES and 22 years old in RPS. (Figure 1)

**Figure:**
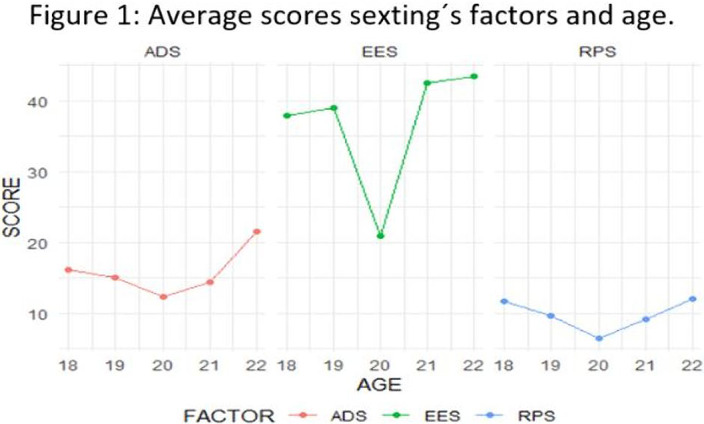

Comparing age and gender, ADS men present higher levels than women, excepting 19 years old. EES and RP, no significant differences are observed. Comparing women’s mean show lower AD levels than men with Cohen’s effect size d = 0.62, (Cohen, 1988). Related to PRS averages, women present lower levels than men without statistically significant differences. Comparing means, women show lower ADS levels than men effecting d Cohen d = 0.46, (Cohen, 1988). (Figure 2)

**Figure:**
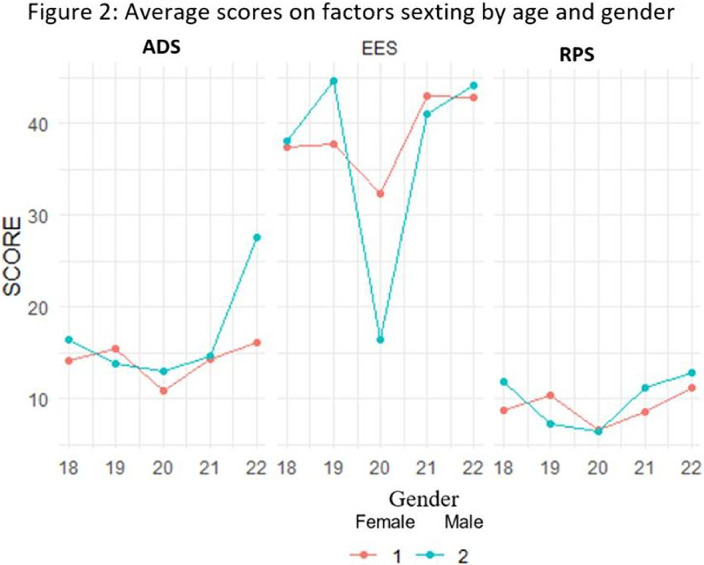

**Conclusions:**

Evidence difference between men and women, in ADS and EES, without pattern associated with age, young men and women sexting

